# Machinability and Energy Efficiency in Micro-EDM Milling of Zirconium Boride Reinforced with Silicon Carbide Fibers

**DOI:** 10.3390/ma12233920

**Published:** 2019-11-27

**Authors:** Mariangela Quarto, Giuliano Bissacco, Gianluca D’Urso

**Affiliations:** 1 Department of Management, Information and Production Engineering, University of Bergamo, Via Pasubio 7/b, 24044 Dalmine, Italy; mariangela.quarto@unibg.it; 2 Department of Mechanical Engineering, Technical University of Denmark, Produktionstorvet, Building 425, 2800 Kgs. Lyngby, Denmark; gibi@mek.dtu.dk

**Keywords:** micro-EDM, zirconium boride, silicon carbide fibers, machinability, advanced material

## Abstract

Several types of advanced materials have been developed to be applied in many industrial application fields to satisfy the high performance required. Despite this, research and development of process suited to machine are still limited. Due to the high mechanical properties, advanced materials are often considered as difficult to cut. For this reason, EDM (Electrical Discharge Machining) can be defined as a good option for the machining of micro components made of difficult to cut electrically conductive materials. This paper presents an investigation on the applicability of the EDM process to machine ZrB_2_ reinforced by SiC fibers, with assessment of process performance and energy efficiency. Different fractions of the additive SiC fibers were taken into account to evaluate the stability and repeatability of the process. Circular pocket features were machined by using a micro-EDM machine and the results from different process parameters combinations were analyzed with respect to material removal, electrode wear and cavity surface quality. Discharges data were collected and characterized to define the actual values of process parameters (peak current, pulse duration and energy per discharge). The characteristics of the pulses were used to evaluate the machinability and to investigate the energy efficiency of the process. The main process performance indicators were calculated as a function of the number of occurred discharges and the energy of a single discharge. The results show interesting aspects related to the process from both the performances and the removal mechanism point of view.

## 1. Introduction

The term advanced ceramics refers to a group of materials achieved starting from inorganic raw materials characterized by a high grade of purity, obtained at high sintering temperatures by means of powder metallurgy technologies. In this way, high-density materials with good mechanical and corrosion characteristics can be obtained. The most important application fields are the realization of medical and dental prosthesis, mechanical components (e.g., ball bearings), extrusion matrix and cutting tools. Some other significant applications are related to high mechanical resistance in critical environments (e.g., gas turbines and heat exchanges), magnetic applications (e.g., insulating material or coating for nuclear reactors) and nanotechnology applications (e.g., nano-turbines) [1,2,3]. A particular group of advanced ceramics is defined as ultra-high-temperature ceramics (UHTCs). UHTCs materials are characterized by excellent performance in an extreme environment and are usually made of borides (ZrB_2_ and HfB_2_), carbides (ZrC, HfC and TaC) and nitrides (HfN). This base structure assigns a high melting point and hardness, relatively good resistance to oxidation and chemical inertness.

During the last years, several studies have argued that the diborides of the group IVb are the most resistant to oxidation and among these compounds, HfB_2_ and ZrB_2_, are the best performing [1,4,5]. Low theoretical density, high melting point over 3000 °C, high electrical conductivity, high fracture toughness and chemical stability in severe conditions make ZrB_2_ a very attractive for applications in critical conditions such as refractories crucible, high-temperature structural components in aerospace, nozzles or armor, passive electronic components for subsystems as the printers, injection systems, the optical and medical devices, actuators and sensors.

Examples of aerospace applications and performances are described in [1,5], which characterize two promising UHTC materials applicable in hot structures for hypersonic and atmospheric re-entry vehicles and a small nose cone have been manufactured and tested at ultra-high temperature. In particular, in [5], they verified the feasibility of a special capsule namely SHARK. This capsule presented an UHTC nose tip manufactured by EDM, and just this tip represented the core of the investigation; in fact, this work identified the instant of the fracture. In the electronics industry, they are applied such as components obtained by laminate and substrate technology. In particular, these special materials can be used for MEMS devices such as pressure transducer and electrostatic actuators. In addition, due to the high wear resistance, ZrB_2_ is a good option for sinking EDM electrodes production [6]. Despite all these characteristics, the application of pure borides is subject to three majors’ limitations. Firstly, the densification process is hindered by the strong covalent bonds; in fact, these materials are characterized by a highly porous structure and very low apparent density, usually about 85% vol. Secondly, ZrB_2_ oxidation generates ZrO_2_, which is characterized by many porosities and therefore non-protective, and B_2_O_3_, which readily evaporates. Thirdly, it is fragile and liable to thermal shock failure. However, the use of pure materials is not enough, especially for structural applications in the high-temperature environment. Thus, to mitigate the shortcoming reported above, the addition of secondary phases, composed of a non-reactive additive like niobium (Nb), disilicides and silicon carbide (SiC), vanadium (V) and carbon (C), is essential and it is the object of many researches [3,7,8,9,10,11,12,13]. The addition of these components allows reducing relative porosity and mechanical and physical properties. Among the additives already studied, SiC is one of the most used and it seems to be the most valuable to enhance the resistance to oxidation by the formation of borosilicate glass, to promote densification by restricting the growth of diboride grains and to lower their sintering temperature [4,6,9]. The double phase materials are usually obtained by a hot pressing process, which, despite the high pressures required (30–50 MPa), allows us to reduce the densification temperature [13,14,15].

Studies related to the microstructure and the characterization of ZrB_2_ sintered with a different compound fraction of silicon carbide (SiC) demonstrated that a high additive fraction reduces the grain size down to 2–3 μm and allows us to increase the flexural strength to 700–1000 MPa. In particular, the addition of 10% vol. of SiC, allows reaching 93.2% relative density. This is enough to limit the grain growth and maximize the strength. Specifically, previous works affirmed that the addition of 20% of SiC generates the best combination of oxidation resistance and mechanical behavior [8,16,17,18,19,20]. Materials characterized by high mechanical properties tend to be really difficult to be processed and ceramic materials are not an exception. Two main categories of machining are effective for processing these kinds of materials: abrasive particle processes (such as grinding, ultrasonic machining and abrasive water-jet) and thermal processes (such as laser ablation and electrical discharge machining) [20]. Today, two main aspects are investigated for ZrB_2_ based ceramics: the application of these materials as electrode and the machining of a ZrB_2_ based ceramics workpiece. In particular, the studies of machining on ZrB_2_ based materials allows increasing process precision and efficiency of these advanced ceramics, and contribute to make the application of ZrB_2_ based ceramics more common [21]. An example of the industrial application of this kind of material is related to micro-nozzles for micropropulsion systems. In [22] a possible application of micro-EDM was explained for manufacturing micro-nozzle with a conical convergent section and a parabolic divergent section. In this work the micro-nozzle was successfully machined, exhibiting high processing efficiency and machining accuracy. As anticipated in the first part of the introduction, another interesting application of ZrB_2_ based ceramic is the nose tip of the sounding hypersonic atmospheric re-entering “kapsule” (SHARK). This tip represents the final part of a re-entering capsule and in the inner part is characterized by a micro-hole where a thermocouple is placed to be protected from the ground impact [5]. This kind of components requires the use of advanced ceramics or advanced materials (for critical environment applications) in combination with features characterized by strict tolerances. In this study, micro-EDM is applied because of the promising results of earlier research in the field [20,23]. Based on the impressive characteristics found in literature, the workpieces are represented by a ZrB_2_ base matrix with the addition of SiC fibers. In this work, the EDM machinability of ZrB_2_ reinforced by SiC fibers is studied with a focus on process stability and process performances. In particular, the effects of a different additive fraction are evaluated. The discharges are characterized, firstly to determine the trigger level for counting discharges, and then to estimate the process performances by verifying the repeatability of the sample distribution. The surface aspect is evaluated by SEM images and by the estimation of surface roughness. All these aspects allow us to identify the main differences with the ED-machining on metal materials and to define if the material removal mechanism is characterized by a different approach.

## 2. Materials and Methods

### 2.1. Materials

#### 2.1.1. Materials Production

The materials investigated were provided by the ISTEC-CNR of Faenza (Italy) and they were prepared as reported in [24] by hot-pressed ZrB_2_ with different fractions of non-reactive additive to reduce the porosity and to improve their high-temperature mechanical properties. ZrB_2_ Grade B (H.C. Starck, Goslar, Germany) and SiC HI Nicalon-chopped fibers (CNR-ISTEC, Faenza, Ravenna, Italy), Si:C:O = 62:37:0.5, characterized by 15 μm diameter and 300 μm length were used for preparing the composites materials. Table 1 summarizes the main structural, physical and thermodynamic properties of ZrB_2_.

The powder mixtures were ball milled for 24 h in pure ethanol using silicon carbide media; then, the dirtiness was withered in a rotary evaporator (ISTEC-CNR, Faenza, Ravenna, Italy). Hot-pressing was performed in a low vacuum environment (∼100 Pa) taking an induction-heated graphite die and applying uniaxial pressure equal to 30 MPa during the heating and increased up to 50 MPa at 1700 °C. The maximum value of the sintering temperature was fixed taking into account the shrinkage curve and a free cooling followed. Table 2 shows the relative density of the samples taken into account.

#### 2.1.2. Materials Characteristics

**Figure 1:**
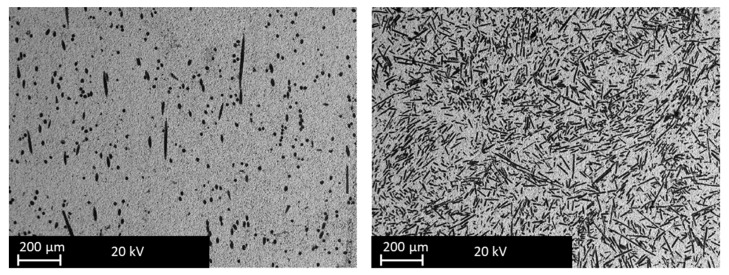
SEM backscatter images of the typical appearance of ZrB5 (left) and ZrB50 (right).

**Figure 2:**
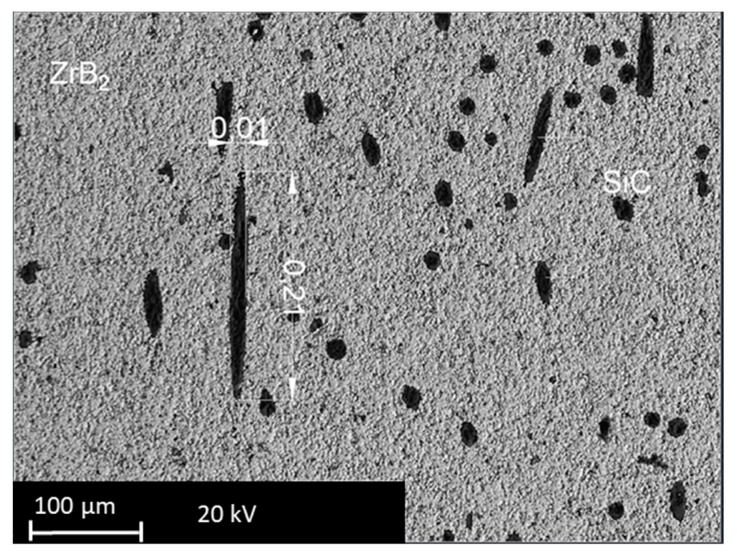
SEM backscatter image showing fibers dimensions.

**Figure 3:**
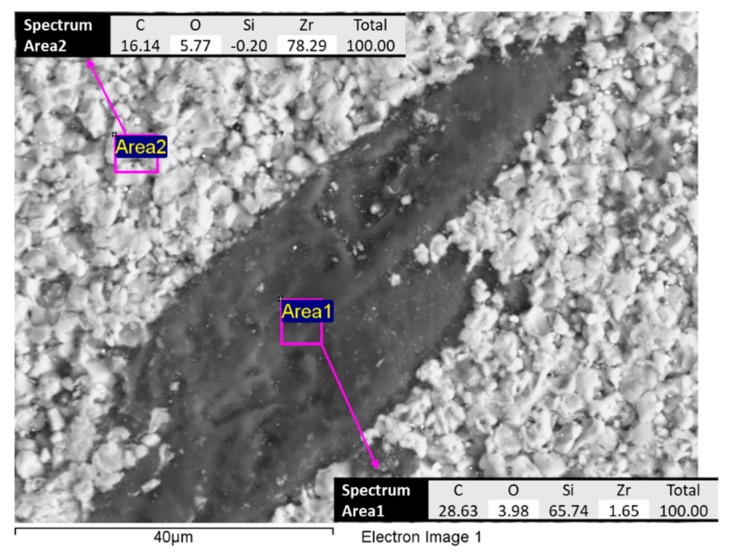
Example of an energy dispersive X-ray (EDX) analysis of the base material ZrB5.

The raw materials were analyzed by a scanning electron microscope (SEM), which allows observing well dispersion of the fibers into the ZrB_2_-base matrix. The fibers are still recognizable as elongated dark structures in backscattered SEM images. Figure 1 reports an example of the typical appearance of ZrB5 and ZrB50. It is possible to notice the different aspects related to the additive fraction. The fibers were distributed homogeneously since no agglomeration was observed. As reported in the literature by [26,27], the fibers tend to align themselves orthogonally to the direction of applied pressure. The fibers inside the base matrix are characterized by a shorter length than one of the starting mixtures. The reduction in length is due to the applied pressure reached during the sintering. In fact, after the preparation, the SiC fibers had dimensions of about 10 μm in diameter and 200 μm in length (Figure 2). Characterization of the as-sintered microstructure by scanning electron microscopy coupled with energy dispersive X-ray analysis (SEM-EDX) was performed. As illustrated in Figure 3, there was a clear separation between the base matrix and the additive, evidence that during the sintering process the base matrix and the additive did not undergo chemical reactions.

### 2.2. Methods

#### 2.2.1. Experimental Set-Up

**Figure 4:**
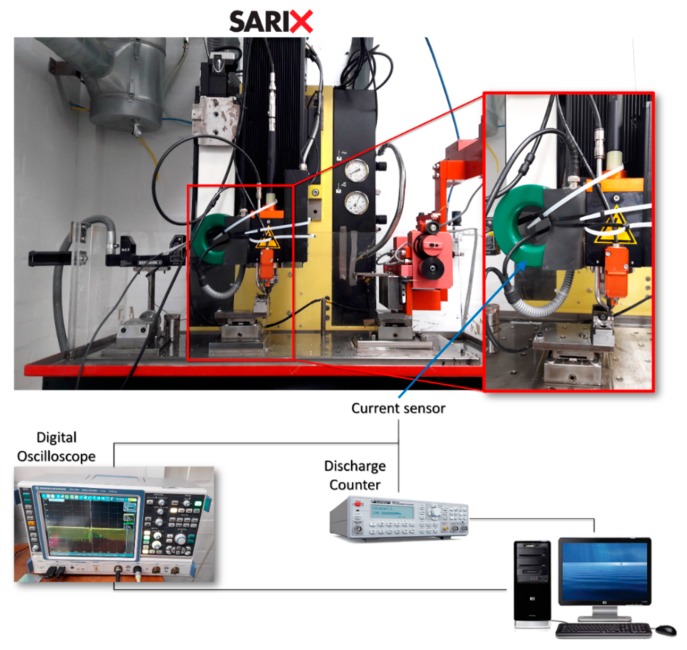
A scheme of the experimental equipment set-up.

In order to explore the EDM machinability of the selected materials, EDM milling tests were carried out with a focus on process stability and process performances. The test feature selected was a circular pocket having a diameter equal to 1 mm and a nominal depth of 200 μm. These micro-features were processed by μEDM milling using a SARIX® SX-200 machine (Sarix SA, Sant’Antonino, Svizzera). Solid tungsten carbide electrodes with a diameter of 300 μm were used as a tool, while the dielectric fluid was hydrocarbon oil. Three different process parameters settings were used to conduct the experimental tests and each of these settings corresponds to a different pulse shape. It is important to remark that in the Sarix EDM machine (Sarix SA, Sant’Antonino, Svizzera), some process parameters such as the peak current and the width are expressed as indexes and the instantaneous values cannot be set because the machine has an autoregulating system. For this reason, the stability and repeatability of the process were investigated by monitoring the discharge characteristics (peak current and discharge energy) and analyzing the distribution of such characteristics during the EDM milling process. This task was performed using a current probe and a voltage probe to measure electric current and voltage during machining. A current monitor with a bandwidth of 200 MHz connected to a Rohde & Schwarz RTO1014 digital oscilloscope (Rohde & Schwarz, Benjamin Franklin Drive, Columbia, MD, USA)was used to acquire the current waveforms. Furthermore, a Hameg HM8123 programmable counter (Rohde & Schwarz, Benjamin Franklin Drive, Columbia, MD, USA) was dedicated to count the occurred discharges. A diagram of the machine set up is shown in Figure 4.

Optimal process parameters were defined for each combination of workpiece material and pulse type and an experimental campaign was performed based on a general full factorial design Table 3). Two factors were taken into account: the additive fraction, defined by five levels, and the pulse type, defined by three levels. Different levels of pulse types identify the different duration of the discharges; in particular, pulse type A is referred to long pulses and high energy per discharge, while pulse-type C identifies short pulses and low energy per discharge. Furthermore, the pulse type is characterized by the different set of process parameters defined by the preliminary tests. Four repetitions were performed for each run [28].

#### 2.2.2. Discharge Population Characterization

**Figure 5:**
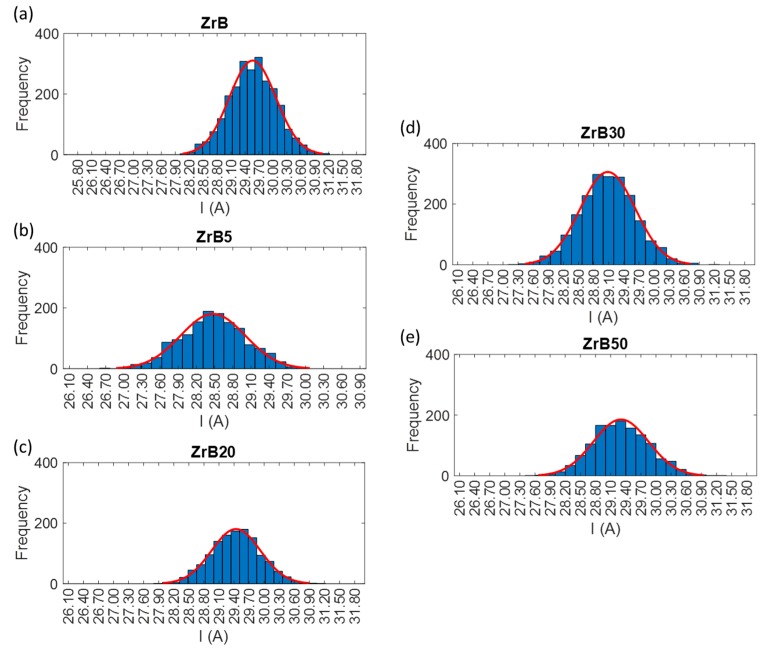
Examples of frequency distribution histograms for pulses occurred during machining performed by Pulse Type A. For the sake of clarity, the y scale was varied among the histograms. (**a**) Tests performed on ZrB sample. (**b**) Tests performed on ZrB5 sample. (**c**) Tests performed on ZrB20 sample. (**d**) Tests performed on ZrB30 sample. (**e**) Tests performed on ZrB50 sample.

**Figure 6:**
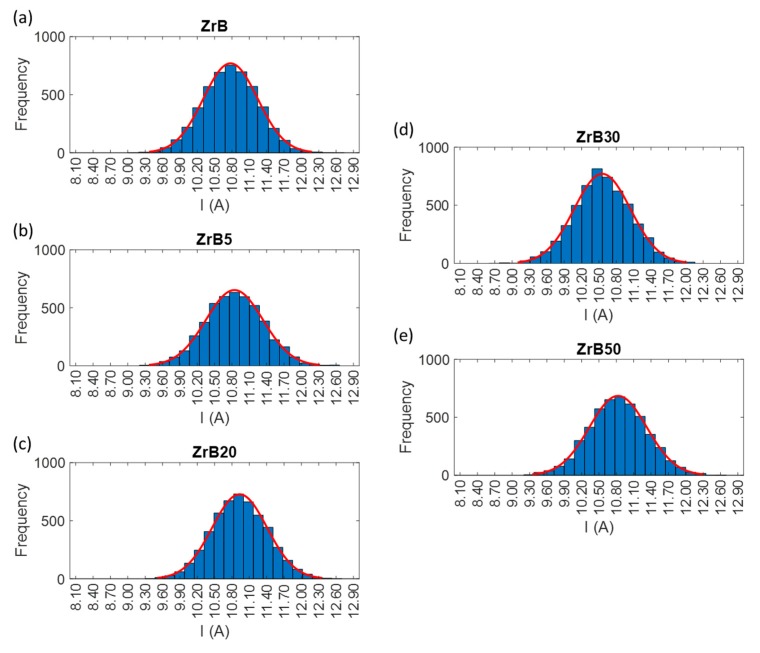
Examples of frequency distribution histograms for pulses occurred during machining performed by Pulse Type B. For the sake of clarity, the y scale was varied among the histograms. (**a**) Tests performed on ZrB sample. (**b**) Tests performed on ZrB5 sample. (**c**) Tests performed on ZrB20 sample. (**d**) Tests performed on ZrB30 sample. (**e**) Tests performed on ZrB50 sample.

**Figure 7:**
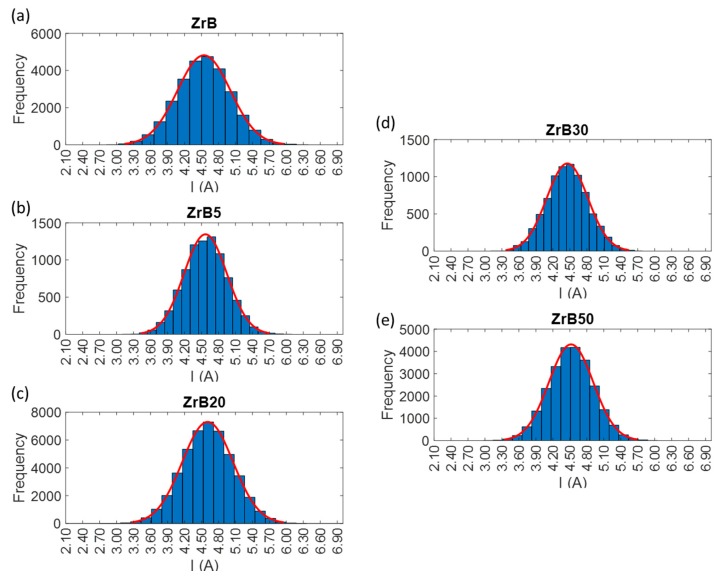
Examples of frequency distribution histograms for pulses occurred during machining performed by Pulse Type C. For the sake of clarity, the y scale was varied among the histograms. (**a**) Tests performed on ZrB sample. (**b**) Tests performed on ZrB5 sample. (**c**) Tests performed on ZrB20 sample. (**d**) Tests performed on ZrB30 sample. (**e**) Tests performed on ZrB50 sample.

In order to determine the trigger level for counting sparks, the discharges population has to be characterized during the EDM process. To do that, the measurement and assessment both of the current and the voltage delivered to the workpiece and the electrode were performed. For this goal, probes dedicated to the current and to the voltage were used. The discharge population is characterized for the purpose of “quantifying” the stability of the process and evaluating the process performances for the machining of the advanced materials selected for this study. The discharge population was characterized by repeated waveform samples of current and voltage signals for the selected pulse types, combined with each material. Waveform acquisition was carried out using a sampling frequency of 40 MSa/s. The trigger level was set-up equal to a very low value for the acquisition process, in order to acquire all the discharge current waveforms, which were stored in the oscilloscope buffer and transferred to a computer. Then, the collected waveforms were elaborated to extract peak current, average discharge voltage, width and energy per discharge. For each waveform sample, the frequency distribution histogram was plotted. Figure 5, Figure 6 and Figure 7 show the distributions of the discharge current for all the combinations grouped according to the pulse type. The histograms provide an overview regarding the waveform frequency as a function of different peak currents. The discharge samples, in some cases, are well represented by a normal distribution. This aspect is typical for conventional materials machined by EDM, but in the application on these advanced ceramic materials, this is not so predictable due to the presence of secondary phase and porosity. Samples distribution is characterized by a good reproducibility and this means that the process is stable. Due to the discharge characterization it was possible to define the explicit value of basic process parameters such as peak current and width. At the same pulse type, for all materials, the peaks have intensities included in similar ranges, while the frequency of the different peaks varies. The pulse properties for each set of process parameters applied in the experiments are reported in Table 4.

#### 2.2.3. Characterization Procedure

A 3D characterization of the micro-slots was performed by a confocal laser scanning microscope (Olympus LEXT, Southend, Essex, UK) with a magnification of 20×. After the data acquisition by the laser microscope, the images were analyzed with a scanning probe image processor software (SPIP, 6.7.3, Image Metrology, Lyngby-Denmark). A plane correction was performed on all the images to level the surfaces prior to assessing the geometrical elements.

**Figure 8:**
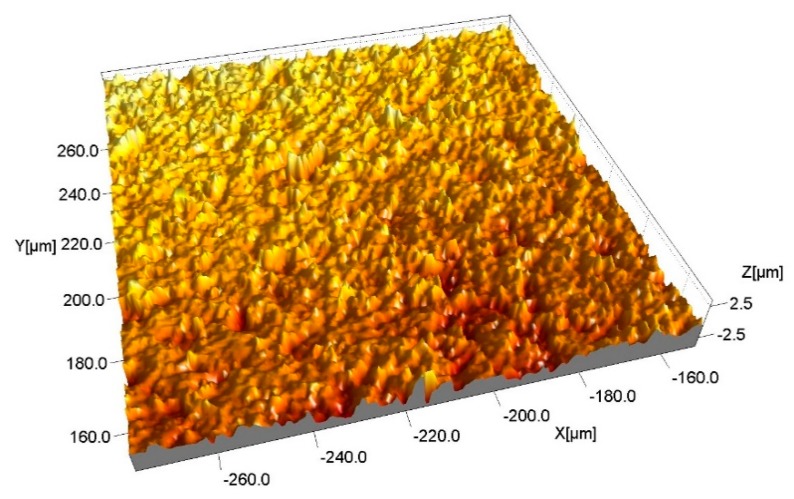
Example of the machined surface.

This software was used to assess the surface roughness (Sa) based on the international standard UNI EN ISO 25178:2017 [29]. Figure 8 shows an example of the texture of a machined surface portion.

Tool wear per discharge (TWD), material removal per discharge (MRD) and tool wear ratio (TWR) were selected as the main performance criteria for the process evaluation. TWD (Equation (1)) was calculated as the ratio between the material removed from the electrode (MRT (mm^3^)) and the number of discharges (N) recorded. The length of the tool wear was measured through a touching procedure executed in a reference position: the length of the electrode was measured before and after the single EDM milling operation. The electrode wear volume was estimated starting from the tool wear length, considering the electrode as a cylindrical part.

(1) $$\mathrm{TWD}=\frac{\mathrm{MRT}}{N\;}.$$

MRD (Equation (2)) was calculated as the ratio between the material removed from the workpiece (MRW (mm^3^)), estimated from the confocal microscope measurements as the volume of micro-cavities, and the number of discharges (N), recorded during the machining of each cavity.

(2) $$\mathrm{MRD}=\frac{\mathrm{MRW}}{N}.$$

TWR (Equation (3)) was calculated as the ratio between TWD and MRD.

(3) $$\mathrm{TWR}=\frac{\mathrm{TWD}}{\mathrm{MRD}}=\frac{\mathrm{MRT}}{\mathrm{MRW}}.$$

Finally, the machined surfaces were observed by scanning electron microscopy (SEM). These qualitative observations allowed analyzing the microscopic aspects and identifying the possible presence of imperfection related to the process.

## 3. Results and Discussion

**Figure 9:**
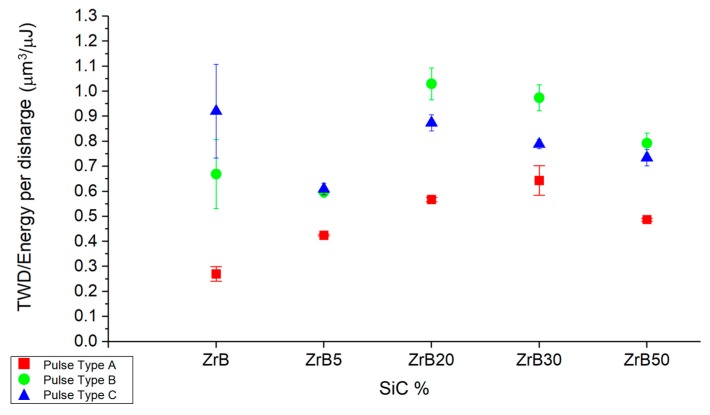
Average ratio between tool wear per discharge (TWD) and energy per discharge as a function of the additive fraction and pulse type.

Figure 9 shows the tool wear per discharge (TWD) divided by the energy of single discharge, as a function of the additive fraction and the pulse type.

The energy efficiency of TWD is lower for the pulse type A. This is a positive aspect because it indicates less impact on the electrode wear. It is possible to observe a common trend as a function of the additive fraction, an exception is represented by ZrB, which presents a large data scatter. The high variation in machining carried out on ZrB could be related to the high level of porosity, which generates an unstable and less repeatable process.

To evaluate the MRD in the correct way, the volume of the micro-slots was adjusted considering the relative density (δ) defined in Table 2. The ${\mathrm{MRD}}_{\delta }$ estimated as reported in Equation (4) was considered for the analysis.

(4) $${\mathrm{MRD}}_{\delta }=\frac{\mathrm{MRW}\cdot \delta }{N}=\mathrm{MRD}\cdot \delta .$$

**Figure 10:**
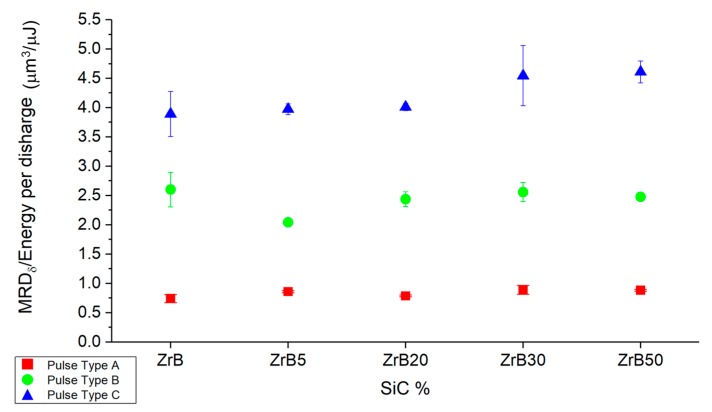
Average ratio between material removal per discharge (MRD) and energy per discharge estimated considering the relative density, the additive fraction and pulse type.

Taking into account the δ allows compensating for the presence of porosity in the sample structure. Considering the energy for single discharge efficiency, the best results were obtained for pulse-type C. The single discharge is characterized by lower duration and energy but the efficiency is higher for all the additive fractions, such as reported in Figure 10. In general, the scatter is low for all conditions and, considering the same pulse type, the MRD_δ_ is characterized by a low variation changing the additive fraction.

**Figure 11:**
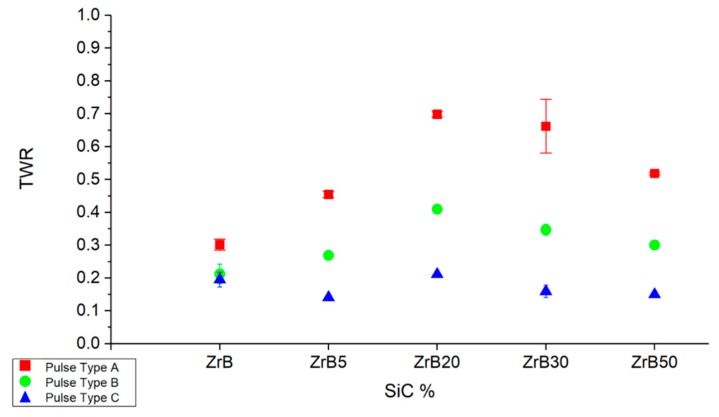
Average tool wear ratio (TWR) as a function of the additive fraction and pulse type.

Figure 11 shows that the TWR for pulse-type A is more sensitive to the additive fraction. In general, the results can be considered stable and repeatable for each condition; in fact, there is low data scatter.

The analysis of the surface roughness (Sa) identifies a strong correlation between the pulse type and the surface finishing. Longer pulses generate low-quality surfaces. In the plot is reported also the relative density of the specimens and it is possible to identify a correlation: high values of relative density generate surfaces with low Sa for all the pulse types on all the samples.

**Figure 12:**
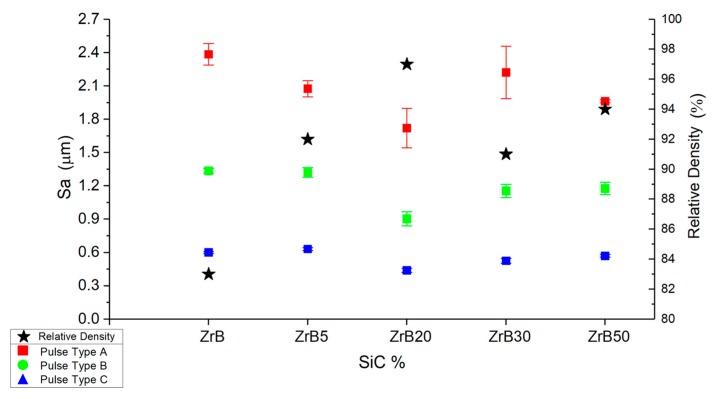
Average values and standard deviation of surface roughness (Sa).

**Figure 13:**
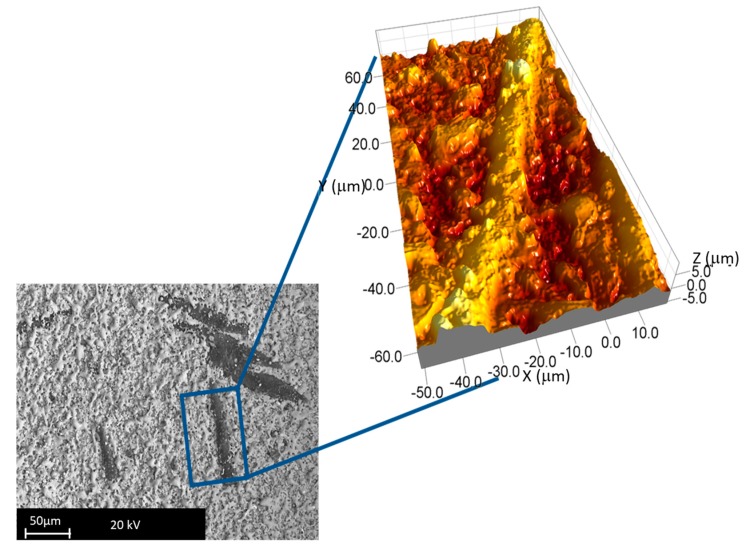
Details of a SiC fiber (backscattering magnification 500×–3D reconstruction magnification 100×).

Figure 12 and Table 5 show higher data scatter for Sa obtained on surfaces machined by pulse type A than for other pulse types. In general, surface quality tends to improve when the porosity level decreases. This aspect is underlined by the introduction of the relative density values reported on a second scale. The plot points out how the increase in the relative density leads to a reduction in surface finishing. The machined surfaces characterized by better surface finishing are obtained on the ZrB20. The surface roughness parameters underline the presence of irregularities on the surfaces; in fact, both the root mean square height (Sq) and the maximum height are characterized by high values. Furthermore, this aspect is supported by the images collected at the scanning electron microscope. Observing the SEM images and correlating them to the 3D reconstruction, a particular aspect can be noted: the SiC fibers, after machining, are characterized by higher-profile height with respect to the ZrB_2_ matrix. Fibers seem like a protrusion on the surface (Figure 13).

**Figure 14:**
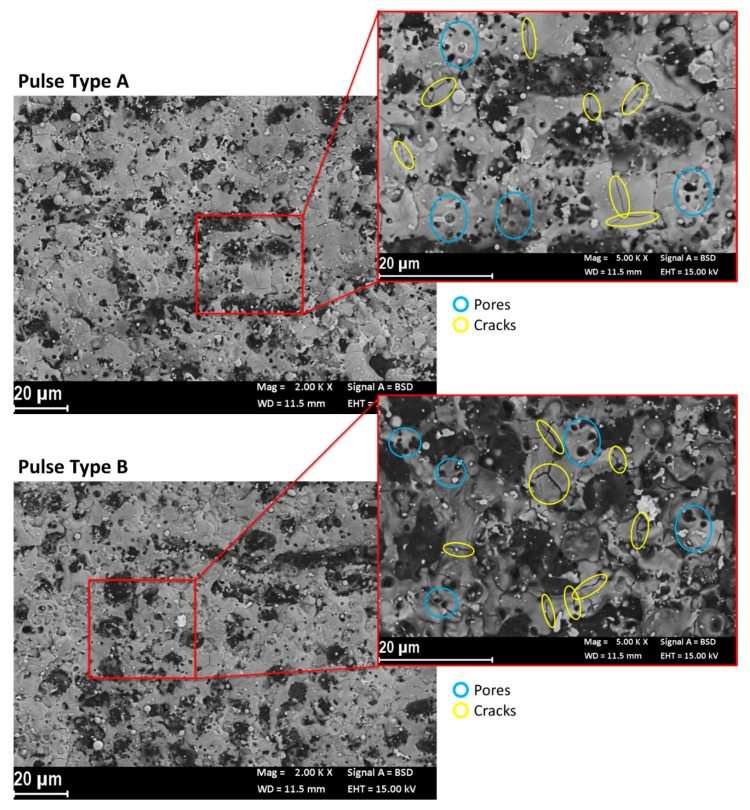

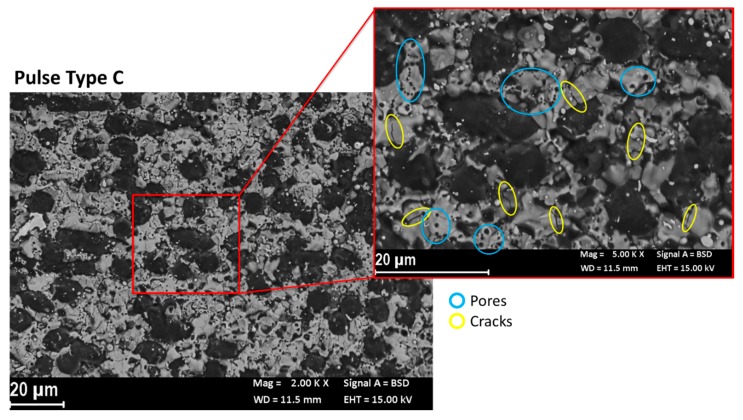
SEM backscatter image of the machined surface. An example of micro-cracks and evaluation of pores area generated during the process. (**a**) Machined surface by pulse type A. (**b**) Machined surface by pulse type B. (**c**) Machined surface by pulse type C.

Another aspect identified thanks to the backscatter SEM images is the surface texture. The images show a non-uniform aspect of the recast layer. This represents a relevant difference comparing these surfaces to the metal ones. In fact, metal workpieces (e.g., stainless steel or aluminum) usually show a uniform structure on surfaces with well-defined craters. On these ceramic materials, the texture shows some microcracks and pores. Figure 14 shows some examples of the topography of machined surfaces. The pores are probably related to the formation of gas bubbles and their “explosion” during the removal process. The dimensions of the pores, in terms of area and numerosity, are characterized by a high level of variability; in particular, shorter pulses generate less and smaller pores. The microcracks morphology is comparable to the typical structure, which indicates the occurrence of an oxidized surface. This aspect might be related to a hot oxidation process or chemical oxidation due to the presence of oxygen in the dielectric medium. It is not to underestimate the possibility of a chemical oxidation process enhanced by the high temperature reached during the EDM process in the machined area. The microcracks formed as a result of an oxidation process are created due to the different thermal coefficients, which characterize the oxidized surface and the raw workpiece material. This means that the microcracks affect only the thickness of the recast layer. Then, these microcracks do not impact on fatigue resistance during the activity of the produced parts. The oxide is more fragile than the base material and then, in the worst case, the oxide could detach from it. Further investigation could involve the analysis of the structure and the inner part of the microcracks by means of the micro-CT scan. The extension of the recast layer is reduced by short pulses; in fact, on the surfaces processed by pulse type C, it is possible to clearly identify the SiC fibers, which are difficult to be observed on surfaces machined with long pulses.

All these observations suggest that this kind of material is not affected by the typical EDM material removal mechanism. The protrusion of the SiC fibers suggests that these parts are not affected by the sparks because of their low electrical conductivity property. Despite the presence of microcracks and pores, there are no fracture surfaces; therefore, it is possible to exclude a mechanical removal mechanism. The available material removal mechanism models of EDM are focused on the sparks erosion with melting and vaporization of workpiece material. Anyhow, in EDM of low conductive materials, the removal mechanism is not well-known and the main hypothesis suggests random spalling, oxidation and decomposition due to alternating thermal stress. This is in agreement with the literature outcomes [30,31].

## 4. Conclusions

A machinability evaluation of advanced ceramic materials composed by ZrB_2_ based matrix hot-pressed with different fractions of non-reactive additive (SiC) was performed in this work. The stability and repeatability of the μEDM process were investigated to identify if the additive fraction influences the process results.

The first step was the characterization of discharges to feature the different pulse types. In this way, it was possible to:

- Define the real value of process parameters such as peak current, pulse duration and energy of each discharge;
- Analyze the discharges distribution highlighting the good reproducibility and stability of the performed tests.

Process performance was evaluated taking into account the energy per discharge to analyze the energy efficiency of the process. Both for the TWD and MRD analysis, the short pulses resulted to be the ones with higher energy efficiency. Low energy per discharge had a higher impact on the material removal rate on both tool and workpiece. The high energy efficiency was positive from the MRD point of view; on the contrary, for the TWD point of view, it was a negative result because it involved higher electrode wear.

In general, there was not a sample with better results than others in terms of overall process performance. An exception could be the ZrB, which was characterized by high scatter data, so the performance was not so repeatable as for other samples. Considering the surface roughness, ZrB20 presents an improvement in the surface quality in comparison to the other materials. This aspect was probably related to the low level of porosity and the high mechanical properties typical of these materials.

The machined surface topography demonstrates that the EDM process on UHTCs materials was characterized by different material removal mechanisms with respect to the EDM machining of well-known materials (e.g., metals). Not only melting and vaporization processes, but also other phenomena, such as oxidation, were involved in the removal of material. The typical phenomena of melting and vaporization were characterized by a lower efficiency because of the high melting temperature and the presence of non-electrically (or low-electrically) conductive parts in the workpiece.

Formation of cracks in the ZrB matrix were observed for pulses type A and B, characterized by higher energy content. For pulses type C, with lower energy content, cracks were not observed. The formation of cracks during EDM processing of the investigated materials generated concerns when these were to be subjected to cyclic loading conditions in operations, particularly if involving tensile loads. The results of the present investigation clearly show that, in such cases, a finishing pass with low energy pulses is a mandatory step to ensure structural integrity. The fatigue performance of such materials as a function of the process parameter combinations shall be characterized.

## Figures and Tables

**Table 1:** ZrB_2_ characteristics [11,15,25].

Property	ZrB_2_	
Crystal system space group	Hexagonal	
Density	6.119	g/cm^3^
Melting temperature	3245	°C
Young’s modulus	489	GPa
Hardness	23	GPa
Coefficient of thermal expansion	5.9 × 10^−6^	K^−1^
Heat capacity at 25 °C	48.2	J/(mol · K)
Electrical conductivity	1.0 × 10^7^	S/m
Thermal conductivity	60	W/(m · K)

**Table 2:** Relative density of the raw materials considered.

Materials	Relative Density (δ)	Sample Name
ZrB_2_	~83%	ZrB
ZrB_2_ + 5% SiC	~92%	ZrB5
ZrB_2_ + 20% SiC	~97%	ZrB20
ZrB_2_ + 30% SiC	~91%	ZrB30
ZrB_2_ + 50% SiC	~94%	ZrB50

**Table 3:** General full factorial design.

	Factors	
	Additive Fraction	Pulse Type
**Levels**	0	
	5	A
	20	B
	30	C
	50	

**Table 4:** Details of pulse type.

Material	Pulse Type	Peak Current (A)	Open Circuit Voltage (V)	Width (μs)	Energy per Discharge (μJ)
**ZrB**	**A**	29.56	150	0.72	792.16
	**B**	10.78	130	0.28	101.84
	**C**	4.81	170	0.06	14.64
**ZrB5**	**A**	28.61	150	0.69	844.39
	**B**	10.84	130	0.32	152.42
	**C**	4.57	170	0.06	17.60
**ZrB20**	**A**	29.44	150	0.70	779.36
	**B**	10.93	130	0.33	150.30
	**C**	4.61	200	0.06	14.73
**ZrB30**	**A**	29.09	150	0.67	784.99
	**B**	10.56	130	0.32	161.00
	**C**	4.45	200	0.06	21.41
**ZrB50**	**A**	29.15	150	0.69	714.13
	**B**	10.82	130	0.33	147.64
	**C**	4.49	200	0.06	12.15

**Table 5:** Average values of main surface roughness parameters.

Material	Pulse Type	Sa (μm)	Sz (μm)
**ZrB**	**A**	2.385	23.610
	**B**	1.335	15.544
	**C**	0.600	8.898
**ZrB5**	**A**	2.075	22.466
	**B**	1.321	17.590
	**C**	0.631	13.569
**ZrB20**	**A**	1.720	17.763
	**B**	0.904	11.430
	**C**	0.439	7.644
**ZrB30**	**A**	2.221	24.999
	**B**	1.154	17.144
	**C**	0.525	13.538
**ZrB50**	**A**	1.961	27.203
	**B**	1.177	24.310
	**C**	0.570	15.387
